# Strength Properties of Sustainable Mortar Containing Waste Steel Slag and Waste Clay Brick: Effect of Temperature

**DOI:** 10.3390/ma14092113

**Published:** 2021-04-22

**Authors:** Md Jihad Miah, Suvash Chandra Paul, Adewumi John Babafemi, Biranchi Panda

**Affiliations:** 1 Department of Civil Engineering, University of Asia Pacific, 74/A Green Road, Dhaka 1205, Bangladesh; jihad.miah@uap-bd.edu; 2 Department of Civil Engineering, International University of Business Agriculture and Technology, Dhaka 1230, Bangladesh; suvashpl@iubat.edu; 3 Department of Civil Engineering, Stellenbosch University, Private Bag X1, Matieland, Stellenbosch 7602, South Africa; ajbabafemi@sun.ac.za; 4 Department of Mechanical Engineering, Indian Institute of Technology Guwahati, Assam 781039, India

**Keywords:** waste steel slag, waste clay brick, mortar, tensile and flexural strength, elevated temperatures

## Abstract

The use of waste streams for the production of sustainable cement-based materials cannot be overemphasized. This study investigates the feasibility of reusing waste steel slag (WSS) and waste clay brick (WCB) as a replacement for natural sand (NS) in mortar. Numerous studies have reported mainly the compressive strength of concrete/mortar, while limited research is available that focuses on the tensile and flexural strength of mortar, and especially the performance at elevated temperature. Hence, this study investigates the tensile and flexural strength of mortar with three different replacement percentages (0, 50 and 100% by volume of NS) of NS by WSS and WCB at normal temperature (without thermal treatment) and after exposure to elevated temperatures (250, 400 and 600 °C). At ambient condition, both tensile and flexural strength were enhanced as the WSS content increased (76 and 68%, respectively, at 100% WSS). In comparison, the strength increased at 50% WCB (25 and 37%, accordingly) and decreased at 100% WCB (23 and 20%, respectively) compared to 100% NS. At elevated temperatures, both the tensile and flexural strength of mortar mixes decreased significantly at 600 °C.

## 1. Introduction

Over the last few years, a vast amount of research has been conducted on the possibility of using various types of alternative raw materials to replace both cement and natural aggregates in the production of cementitious materials [1,2,3]. This is because of the scarcity and continuous depletion of natural resources. Moreover, concrete structures continue to attain their service life and require demolition, thereby generating tons of waste. These factors have necessitated research on a global scale to reuse waste materials, to minimize their environmental impact and emphasize sustainable development [4]. Additionally, replacing natural aggregates with waste is reducing the CO_2_ footprint.

Steel slag is a by-product mainly generated from converting iron to steel or melting the scrap to make new steel [5]. In optimizing waste from scrap metal, a significant amount of steel slag is also generated. Similarly, a significant amount of clay brick waste is generated from the demolition of masonry buildings and the manufacturing industry after being rejected when off standard [6]. Ultimately, these materials are dumped into landfill or, if economically feasible, used for landscaping purposes [7,8]. In some cases, they are recycled as aggregates in concrete where natural aggregates are scarce [6], and their performance investigated in relation to control samples.

One such study investigated the mechanical properties of mortar made with fine aggregates of electric arc furnace slag at different levels (0, 25, 50, 75 and 100%) [9]. The compressive and flexural strengths of mortar were reported to increase as the slag content in the mix increased. At 90 days of testing, the strengths continued to increase by 25 to 30% compared with the virgin mortar mix made with 100% natural fine aggregates. This improvement in the strength was triggered by the pozzolanic behavior of the fine slag, which reacted chemically with the calcium hydroxide of the cement clinker to form compounds possessing cementitious properties [9]. The effect of steel slag content on the samples of normal- (range 30–35 MPa) and high (range 64–75 MPa)-strength concrete was also reported in [10]. A fluctuating impact was observed for both strength classes with an optimum compressive strength at 20 and 80% for normal-strength concrete. The optimum contents for high-strength concrete were 30 or 100%. The steel slag used was said to more positively impact the normal-strength concrete than the high-strength concrete. It has been shown that steel slag aggregates consist of active C_2_S and C_3_S that can participate in cement’s hydration process and increase the strength [11].

Steel slag as fine powders was also used in mortar to replace cement [8,12]. At the lower level (10%) of cement replacement by steel slag, a 6% increase in compressive and splitting tensile strength was found at the 28 days test [12]. However, at 80% replacement, an approximately 30% reduction in the compressive strength was reported compared to the reference mortar [8]. This reduction was attributed to the low hydraulic reactivity of steel slag at a high content. Additionally, at this replacement level, calcium silicate hydrates (C-S-H) gel, the main hydration product of cement, warps the steel slag particles and reduces the cement particle surface C-S-H gel layer thickness [8], hence, reducing the strength of cementitious materials with a high volume of steel slag [12].

Like steel slag, recycled clay brick powder was also used to produce cementitious materials, and its effect on various rheological, mechanical and durability properties was investigated [3,13,14]. An investigation of the mechanical properties of mortar where clay brick powder replaced natural fine sand at three particle sizes (fine: 0.15–0.30 mm; medium: 0.3–0.6 mm; and coarse: 0.6–4.75 mm) and three replacement levels (10, 20, and 30%) was reported in [3]. The flexural strength showed no noticeable change irrespective of the particle size and content of the brick powder. However, the compressive strength gradually increased at all sizes and replacement levels. The maximum compressive strength was found for mortar with fine- and medium-size brick powder at a 10% replacement level. It was concluded that clay brick powders in the mix provide additional pozzolanic activity, increasing the strength. A similar trend of higher strength than the control at lower brick powder content (5–20%) was also reported [15,16]. At a higher replacement level, brick powder adversely affects mechanical strength. Generally, brick aggregates are porous, and at a higher replacement level, the total pore volume and porosity of the sample increase, thus degrading the strength [15].

In this study, the natural fine aggregates in mortar were partially to fully replaced by the waste steel slag and clay brick aggregates. At present, limited research can be found where the performance of mortar made with these two recycled materials is used to investigate the mechanical properties at elevated temperatures. At elevated temperatures, as in the case of a fire, cement-based materials’ properties could be significantly compromised, thereby affecting the service life of cement-based structures. Two phenomena occur at elevated temperatures: initial loss of moisture from the cement paste resulting in a contraction and after that, an expansion of the paste and other aggregates as the temperature continues to rise, thereby leading to spalling [17]. Some studies showed that incorporating waste glass powder as either cement or natural fine sand substitutes improved residual strength at elevated temperatures [17,18].

Natarajan et al. [19] studied the residual compressive and flexural strength of self-compacting mortar made with different replacement percentages (10, 20, 30, 40 and 50%) of natural sand by glass powder after exposure to elevated temperatures (200, 400, 600, 800, 1000 and 1200 °C) at a heating rate of 20 °C/min. The authors found that the compressive and flexural strength decreased with an increase in temperature, and it was more pronounced for the mortar made with higher glass powder content. This higher reduction in strength was in agreement with the increased porosity at increasing temperatures. The authors stated that the decrease in strength of the mortar exposed to elevated temperatures was due to the mismatch between cement paste and aggregate as the cement paste shrank; in contrast, the aggregate expanded at high temperatures, which induced cracking in the matrix leading to lower strength.

Tran et al. [20] investigated the mechanical strength (compressive, tensile and flexural strength) and microstructure changes of alkali-activated slag mortar after exposure to the temperature range of 200 to 1000 °C. The specimens were cured by different curing methods and were exposed to high temperatures of 200, 400, 600, 800 and 1000 °C at a heating rate of 6.67 °C/min. The mechanical strength of the mortar mixes was reported to decrease as the temperature increased, and a higher reduction was observed at higher temperatures. The strength reduction was due to the cracking in the mortar matrix, confirmed by the Scanning Electron Microscope (SEM) analysis of each mix after exposure to the elevated temperatures [20]. Another study carried out by Tran and Kwon [21] noted that alkali-activated slag mortar was sensitive to elevated temperatures (200, 400, 600, 800, 1000 and 1200 °C), and the reduction in the mechanical strength varied dependent on the percentage of Na_2_O present in the mortar mixes. A higher decline in mechanical strength was observed from 400 to 800 °C for the mortar made with 10% Na_2_O. Likewise, Roy et al. [22] observed that at higher temperatures (>500 °C), the reduction in mechanical strength (compressive strength, elastic modulus, flexural strength and tensile fracture energy) was more for concrete made with electric arc furnace slag fine aggregate than NS due to higher internal cracks and interconnected surface cracks in slag concrete than NS concrete.

For this study, the flexural and tensile strength characteristics of mortar specimens were investigated at ambient temperature and after exposure to elevated temperatures. The outcome of this research may help designers to optimize their cementitious materials with the inclusion of steel slag and clay brick power, which can further help in achieving the circular economy of a country [23].

## 2. Experimental Methodology

### 2.1. Materials and Mix Design

**Figure 1:**
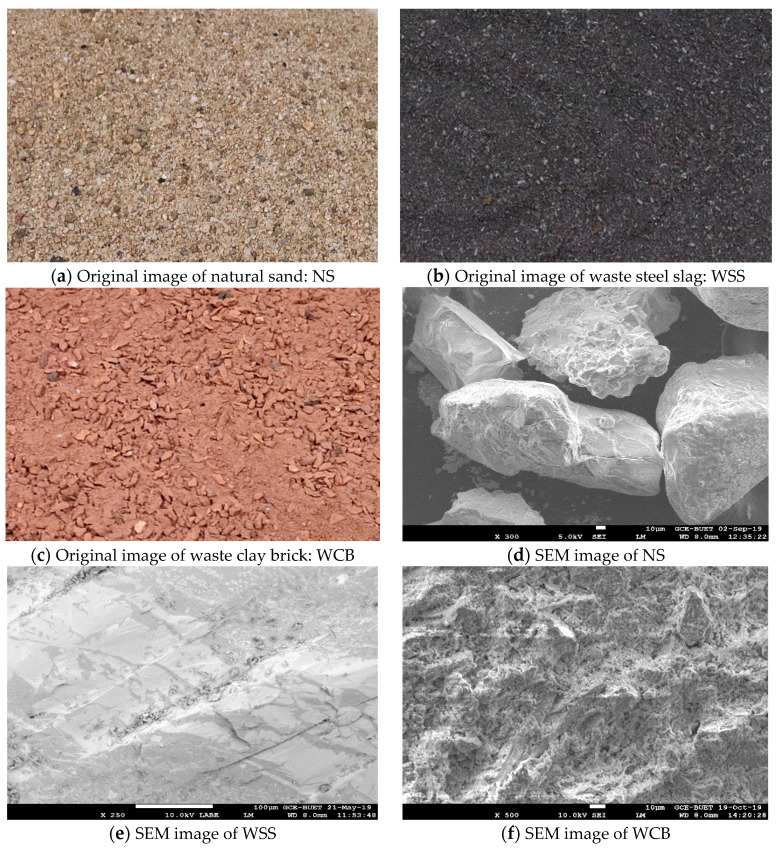
Pictures of natural sand—NS (**a**), waste steel slag—WSS (**b**) and waste clay brick—WCB (**c**), and SEM images of NS (**d**), WSS (**e**) and WCB (**f**), respectively.

The original images of the waste steel slag (WSS) and waste clay brick (WCB) used as replacements for natural sand (NS) are shown in Figure 1a–c. The NS was collected from the local market, which usually comes from riverbeds by digging. In contrast, WSS is the waste powder of induction furnace steel slag, which is itself a by-product of the steelmaking industry produced during the melting of scrap at around 1500 °C. The steel slag boulders were collected from the crushing plant of induction furnace steel slag of a local steel manufacturing company.

The slag boulders were a combination of porous (lightweight with more voids) and dense microstructures (lower voids, i.e., lower porosity). These large-sized slag boulders were manually crushed to smaller sizes for use as coarse aggregate in concrete, and during this process, the WSS was produced. Due to the scarcity of natural stone aggregate, in many countries of South Asia, the concrete industry mainly depends on burnt clay brick aggregate for making concrete. These bricks are produced from wet clay by burning in an open brickfield kiln at around 1150 °C. After burning and cooling, different classes of bricks are produced, such as first class brick (commonly used for making aggregate in Bangladesh), second class, third class and picket bricks. First class bricks were collected from the brickfield and manually crushed at the concrete laboratory to make coarse aggregate. Many fine waste products are produced during crushing bricks—this waste product is used, named WCB. Both WSS and WCB are grown enormously and used as landfilling materials but are hazardous to human health. These two materials were sieved using ASTM standard sieves, and the maximum size of these two materials was 4.75 mm.

The microstructure and surface roughness of NS, WS, and WCB were analyzed through scanning electron microscopy (SEM). The SEM images of NS, WSS and WCB, are shown in Figure 1d–f. It was found that the particle shape of NS was nearly well-rounded, while the WSS and WCB were crushed and had excellent surface roughness.

**Figure 2:**
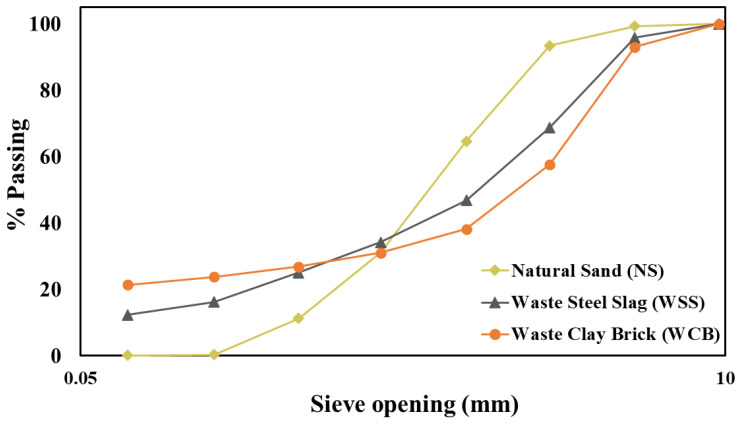
Grading curves of natural sand—NS, waste steel slag—WSS and waste clay brick—WCB.

The grain size distribution of the aggregates (NS, WSS and WCB) was determined using ASTM standard sieves [24], and the results are presented in Figure 2. Though the three materials had different trends, all were well-graded aggregate, as the S-shaped gradation curves found. The maximum size of the NS, WSS and WCB was 4.75 mm. Most importantly, it was found that the WSS and WCB had a significantly higher content of smaller particles (smaller than 74 µm) than NS (Figure 2). A significantly higher amount of very fine particles of WSS and WCB was found on the pan of the ASTM sieve. The amount of materials retained on the sieve number #200 (sieve opening of 74 µm) and pan (no holes, particle size smaller than 74 µm) were 0.20 and 0.12% for NS, 3.86 and 12.38% for WSS and 2.37 and 21.41% for WCB, respectively, of the total amount that was used for sieve analysis. The WSS and WCB contained a higher content of coarser particles than the NS, and the fineness modulus of the materials were 3.0, 3.13 and 3.29, respectively, for NS, WSS and WCB. The fineness modulus of the materials was obtained by summing the total percentages of material in the sample that was coarser than each of the following sieves (cumulative percentages retained; 4.75, 2.36, 1.18, 0.60, 0.30 and 0.15 mm) and dividing the sum by 100 [24].

The specific gravity, absorption capacity and unit weight of the materials were tested as per ASTM C128 [25] and ASTM C 29 [26] standards. The specific gravity, absorption capacity and unit weight were, respectively, 2.56%, 5.86% and 1598.30 kg/m^3^ for NS, 3.24%, 1.0% and 1919 kg/m^3^ for WSS and 1.70%, 13.90% and 1422 kg/m^3^ for WCB. The chemical composition of NS, WSS and WCB were determined using X-ray fluorescence (XRF) analysis, and the results are presented in Table 1. The higher specific gravity of the WSS is in agreement with the higher content of Fe_2_O_3_ (Table 1). CEM II 42.5 N as a binder was used for all the mortar mixes, and the chemical compositions are given in Table 1.

To investigate the impact of WSS and WCB as the replacement for NS on the tensile and flexural performances of mortar at ambient temperature and after exposure to elevated temperatures, three different replacement percentages (0, 50 and 100% by volume of NS) of NS by both WSS and WCB were used. All the mortar mixes were made with a water to cement ratio of 0.50. The mix design of the mortar mixes is summarized in Table 2. 

### 2.2. Experimental Program and Test Procedures

Testing the strength of mortar mixes was performed in two different parts: (i) test without thermal treatment and (ii) test after exposure to elevated temperatures. For all mortar mixes, briquet specimens for tension and prismatic specimens (40 mm × 40 mm × 160 mm) for the flexural test were used as per ASTM 307 [27] and ASTM 348 [28], respectively. The normal temperature test (without thermal treatment) was performed at 28 and 90 days.

**Figure 3:**
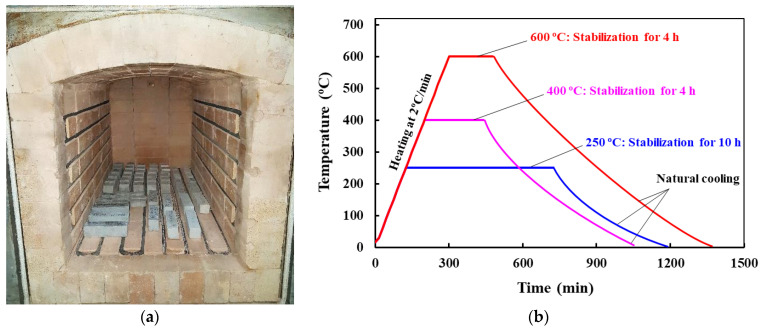
Electric furnace (**a**) and heating profiles of the test specimens (**b**).

The specimens were heated inside the electric furnace (Figure 3a) at a heating rate of 2 °C/min up to the target temperatures of 250, 400 and 600 °C to investigate the effect of elevated temperature on the tensile and flexural strength of mortar. As cement-based mortar/concrete has lower thermal conductivity and diffusivity than other materials like steel, leading to a slower penetration of temperature inside the mortar, prolonged heating was necessary to have a uniform temperature in the specimen [29]. Therefore, after reaching the target temperature, the heating curves were stabilized for 10, 4 and 4 h for 250, 400 and 600 °C, respectively, to ensure homogenous heating all through the mortar specimens [29,30,31]. Later, the specimens were cooled down to ambient temperature (20 °C) inside the closed furnace naturally by switching off the electric furnace. The heating and cooling curves for all temperatures are shown in Figure 3b. The residual tensile and flexural tests were performed at room temperature.

It should be noted that the heating of the mortar specimens was conducted at 90 days of curing. As blended cement (CEM II 42.5 N) was used for all mixes, consisting of 80–94% clinker, 6–20% of mineral admixture (slag and fly ash) and 0–5% of gypsum, the hydration of binder might not have been completed. Therefore, 90 days for the heating test was chosen to study the stabilized main properties of concrete/mortar (e.g., strength). In contrast, the control specimens of all the mixes were not exposed to thermal treatment and tested at ambient temperature (20 °C). However, before and after each heat treatment, the mortar specimens were weighed to determine the density and mass loss of specimens.

## 3. Results and Discussion

### 3.1. Tensile and Flexural Strength at Normal Temperature

**Figure 4:**
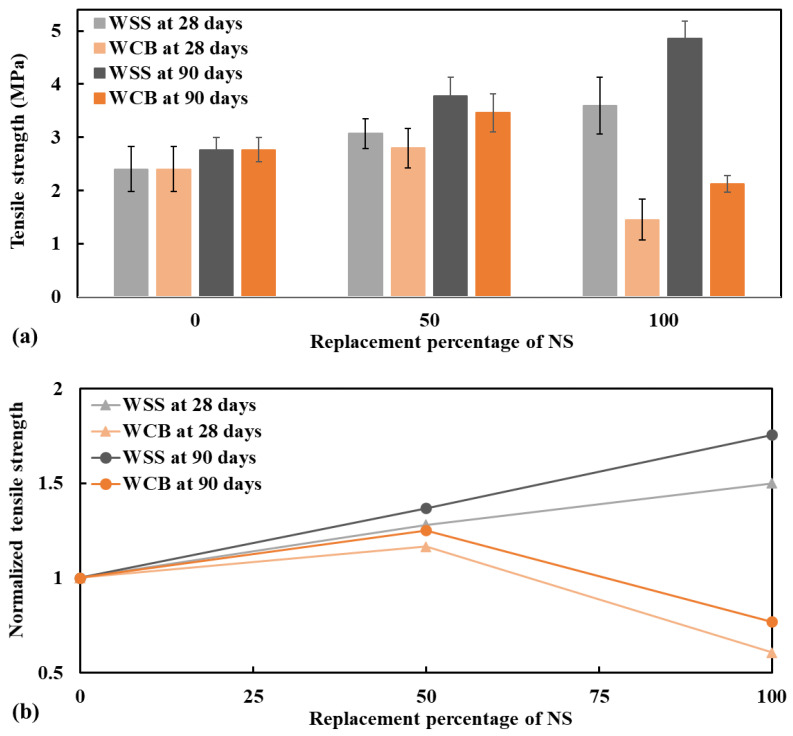
Tensile strength (**a**) and normalized tensile strength (**b**) of mortar specimens performed at 28 and 90 days, respectively.

**Figure 5:**
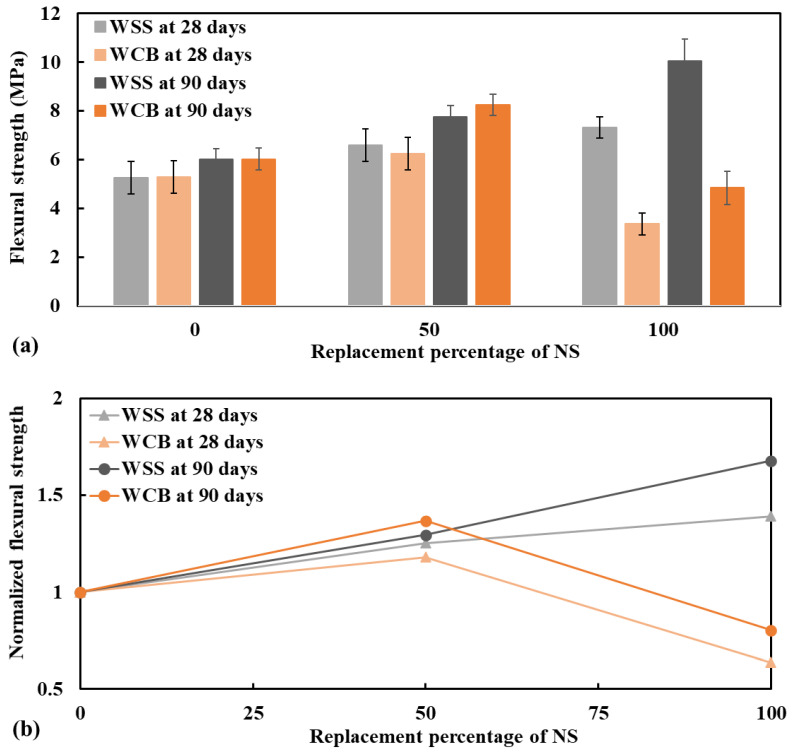
Flexural strength (**a**) and normalized tensile strength (**b**) of mortar specimens tested at 28 and 90 days, respectively.

The tensile and flexural strength at 28 and 90 days are presented in Figure 4 and Figure 5. Both tensile and flexural strength of mortar made with WSS increased as the replacement level of NS increased. In contrast, the strength increased at 50% WCB but decreased at 100% WCB. The increase in strength of mortar made with 50 and 100% WSS were 28 and 50% (Figure 4b), respectively, for the tensile strength and 25 and 39% (Figure 5b), accordingly, for the flexural strength at 28 days with respect to the control specimen (mortar made with 100% NS).

Moreover, the increase in strength at 50% WCB was 16 and 18%, respectively, for the tensile and flexural strength at 28 days compared to the control specimen. This higher tensile and flexural strength of mortar made with WSS and WCB could be attributed to the crushed coarser particles, excellent surface roughness and more angular shape than NS (Figure 1b,c,e,f). These properties provide better interlock among the particles and a more robust interfacial transition zone (ITZ) around the aggregate, resulting in higher tensile and flexural strength.

Conversely, the particle shape of NS was uncrushed, smooth in surface and nearly well-rounded (Figure 1a,d), which provided a weak bond among the particles and weak ITZ between NS and cement paste, leading to lower tensile and flexural strength. Similar results were reported in the literature [32]. It is noted that a significantly higher amount of very fine particles (smaller than 74 µm) of WSS (12.38%) and WCB (21.41%) was found on the pan of the ASTM sieve. These very fine particles are expected to fill the microvoids in the mortar matrix, which significantly reduces the mortar mixes’ porosity and strengthens the ITZ by filling the void at the interface between cement paste and aggregate, i.e., dense microstructure. Perhaps, this lower porosity of the mortar would provide higher tensile and flexural strength of the mortar mixes.

The decrease in tensile and flexural strength at 100% WCB could be due to the higher void/porosity in the mortar mix induced by a higher content of coarser particles (Figure 1c) and the lower mechanical strength and modulus of elasticity of WCB compared to NS (WCB is more porous than NS, Figure 1d,f). The lower tensile and flexural strength of the mortar also could be due to the higher water absorption capacity of WCB than NS (13.90% for WCB and 5.86% for NS), which provides weak ITZ by absorbing water from its surrounding cement paste and increases the un-hydrated cement particles in the mortar mix, thus offering lower tensile and flexural strength. 

An important observation is that the mortar made with WSS and WCB had better performance at the extended curing age (at 90 days) than the control specimen. Both tensile and flexural strength of mortar made with WSS and WCB were increased at 90 days over NS. The increase in the strength of mortar made with 50% WSS and 100% WSS was 37 and 76%, respectively, for tensile strength and 29 and 68%, respectively, for flexural strength. While the tensile and flexural strength increased at 50%, WCB was 25 and 37%, respectively. This higher strength of mortar made with WSS and WCB at 90 days could be explained by the micro-filler effect and the activation of the pozzolanic reaction of the WSS and WCB particles.

**Figure 6:**
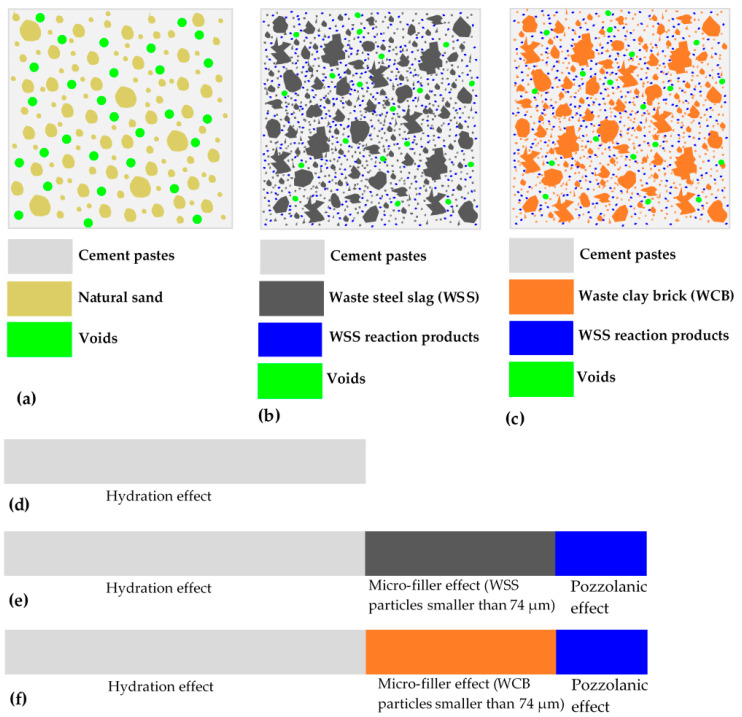
Conceptual diagram of the cross-section of the mortar made with 100% NS (**a**), 100% WSS (**b**) and 50% WCB (**c**) and the effect of cement hydration, micro-filler and pozzolanic action for 100% NS (**d**), 100% WSS (**e**) and 50% WCB (**f**) on the development of strength of mortar, respectively (dimensions of the objects in the figures are not actual).

To understand the mechanism further, conceptual diagrams of the cross-section of the mortar made from 100% NS, 100% WSS and 50% WCB were drawn and are presented in Figure 6. The development of strength could occur in three steps: (i) hydration effect, (ii) micro-filler effect and (iii) pozzolanic effect. As the NS did not contain (or contains a very small quantity of) particles smaller than 74 µm (i.e., particles passed through the sieve number #200 (the opening of which is 74 µm); however, it is noted that sieves with openings smaller than 74 µm were not used, and materials were collected on the pan), the hydration effect mainly contributed to the development of strength, which was more effective up to 28 days, and no micro-filler and pozzolanic effect existed (Figure 6a,d). Regarding the mortar made with 100% WSS and 50% WCB, as previously mentioned, the WSS and WCB contained a significantly higher content of very fine particles (smaller than 74 µm), which filled the microvoids (i.e., lower porosity). Hence, the strength development due to the hydration effect and micro-filler effect contributed to the strength. Moreover, as the particle sizes of WSS and WCB are very small, they might have sufficient specific surface area to permit the pozzolanic reaction (Figure 6b,c,e,f). Therefore, WSS and WCB might react with the hydrated products of the clinker (i.e., calcium hydroxide—Ca(OH)_2_) in the presence of water in the mortar matrix and then form secondary calcium silicate hydrate (CSH) gels (Ca(OH)_2_ + WSS/WCB fine particles + H_2_O = CSH). As a result, the higher content of CSH gels would fill the micropores, strengthening ITZ, leading to the higher tensile and flexural strength of the mortar mixes.

The results presented in this study reveal that the mortar made with WSS increased in strength more than the mortar with WCB (due to the dense microstructure and higher strength of WSS than WCB, Figure 1e,f) and found that WSS can entirely replace NS, while WCB can only replace 50%. Therefore, it is recommended to use up to 100% WSS and 50% WCB as a replacement of NS for mortar production without compromising the mechanical strength, which reduces the demand for NS, uses eco-friendly materials and provides environmental solutions by resolving the disposal problem.

### 3.2. Dry Density and Mass Loss after Exposure to Elevated Temperatures

**Figure 7:**
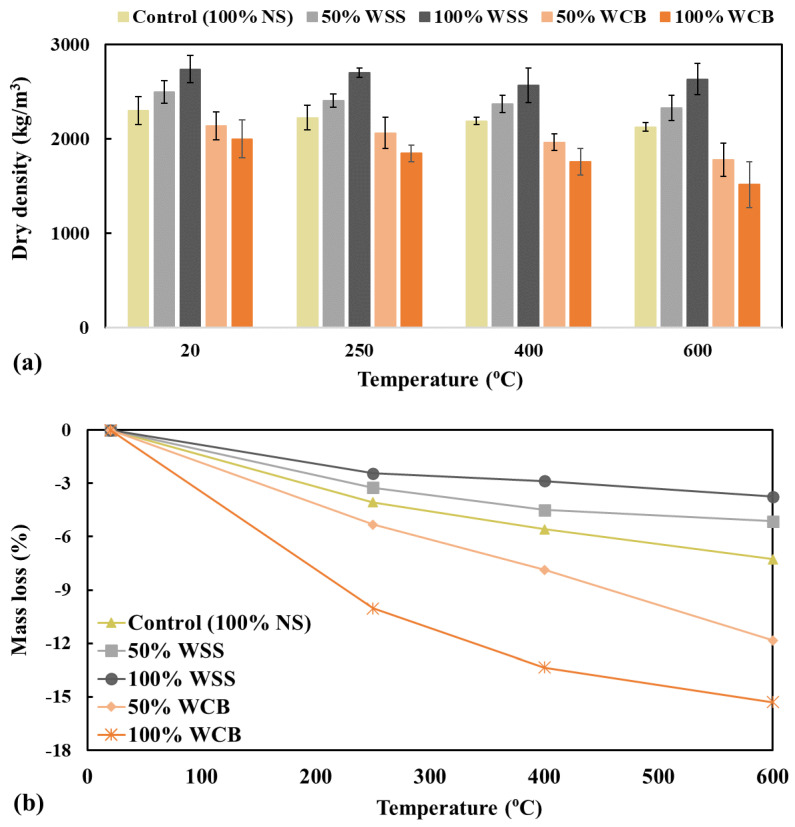
Dry density (**a**) and mass loss (**b**) of the mortar mix after exposure to elevated temperatures.

The average dry density of the hardened mortar mixes at normal temperature and after exposure to elevated temperatures is shown in Figure 7a. The test at normal temperature (without thermal treatment, i.e., 20 °C) shows that the mortar made with WSS had a higher density than NS and WCB. The average density of mortar mixes made with 100% NS, 50% WSS, 100% WSS, 50% WCB and 100% WCB were 2300, 2498, 2742, 2137 and 2000 kg/m^3^, respectively. The maximum increase and decrease were observed at 100% WSS and 100% WCB, respectively, approximately 19% higher for 100% WSS and 13% lower for 100% WCB than the control specimen.

This behavior is directly linked with the specific gravity and unit weight (2.56 and 1598.3 kg/m^3^ for NS, 3.24 and 1919 kg/m^3^ for 100% WSS and 1.70 and 1422 kg/m^3^ for 100% WCB) of the materials, the specific gravity and unit weight of which increased by 27 and 20%, respectively, for WSS and decreased by 34 and 11%, respectively, for WCB compared to NS. As the temperature increased, the dry density of all the mortar mixes decreased, and the decrease in density was more significant at higher temperatures (e.g., 400 and 600 °C). The dry density after exposure to 600 °C of the mortar mix made with 100% NS, 100% WSS and 100% WCB were 2129, 2635 and 1518 kg/m^3^, respectively.

The mass loss of the specimens was measured by weighing the mass before and after heat treatment of all mortar mix specimens, and the results are presented in Figure 7b. Significantly higher mass loss was observed for the mortar made with WCB than NS and WSS. The maximum loss was observed for the higher temperature (e.g., 600 °C) and the mortar made with 100% WCB. This behavior could be linked to the higher temperature, which accelerates water evaporation (more and rapid drying) and thermal damages (i.e., higher crack (Section 3.3), which allows the higher release of moisture from the specimens [31,32]). Compared with control specimens, the minimum mass loss was observed for the mortar made with 100% WSS, while a noticeable mass loss was found for 100% WCB for all thermal loads. The mass loss of mortar mixes made with 100% NS, 100% WSS and 100% WCB after exposure to 600 °C were −7.27, −3.75 and −15.30%, respectively. These results agree with the dry density and the temperature and absorption capacity of the materials (5.86% for NS, 1.0% for WSS and 13.90% for WCB). This higher absorption capacity and higher porosity due to the higher content of coarser particles and the more porous microstructure (Figure 1f) of WCB than NS and WSS could be the main reason for the higher mass loss of the specimen at higher temperatures. Indeed, the higher the water available in the specimens, the higher the mass loss that occurred. The higher available water would also induce higher pore pressure [30] (i.e., higher tensile stress), resulting in higher thermal cracks and more water release from the specimens during heating, leading to the higher value of the mass loss.

### 3.3. Tensile and Flexural Strength after Exposure to Elevated Temperatures

**Figure 8:**
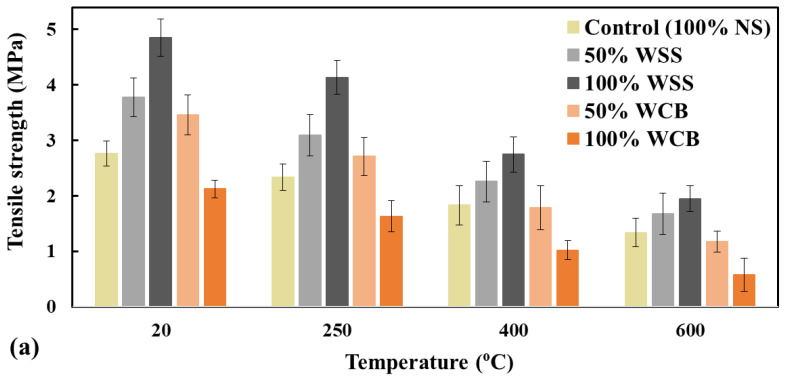

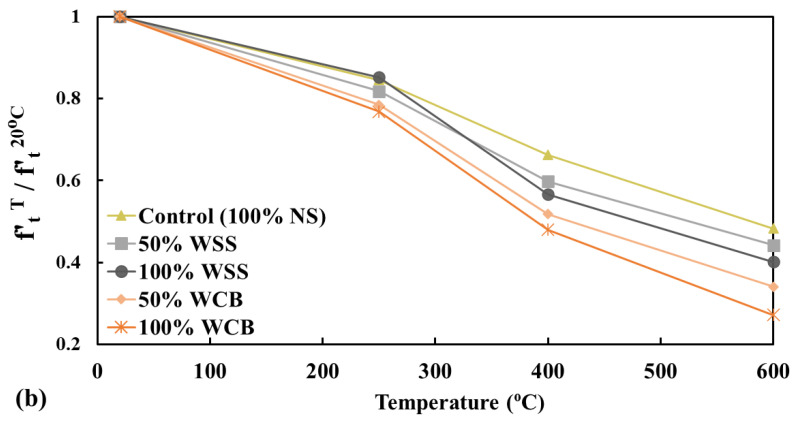
Tensile strength—$f_{t}^{\prime }$ (**a**) and normalized $f_{t}^{\prime }$ (**b**) of mortar mixes tested at room temperature after exposure to elevated temperatures.

**Figure 9:**
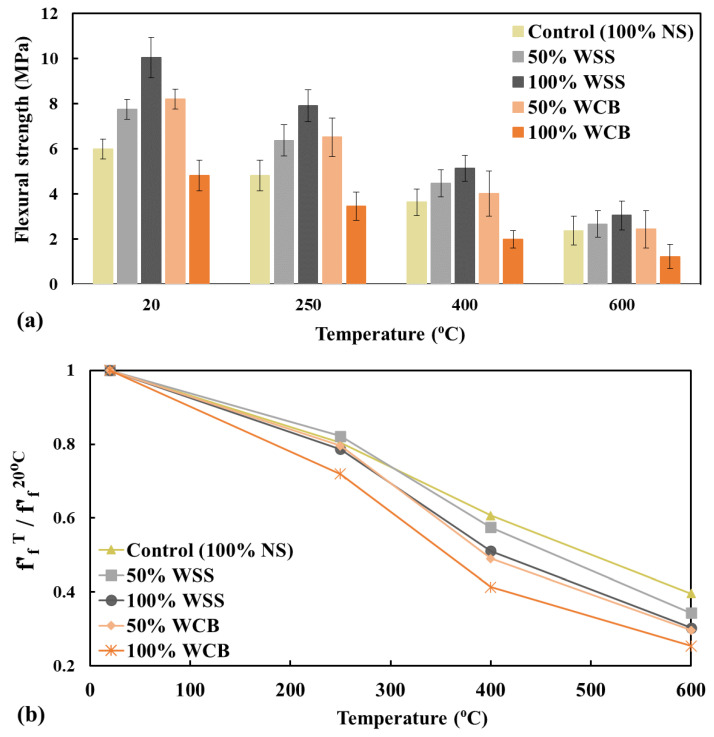
Flexural strength—$f_{f}^{\prime }$ (**a**) and normalized $f_{f}^{\prime }$ (**b**) of mortar mixes performed at room temperature after exposure to elevated temperatures.

The residual tensile and flexural strength of mortar mixes after exposure to elevated temperatures are presented in Figure 8 and Figure 9. It is shown that both the residual tensile and flexural strength of mortar was decreased with the increased temperatures, and a more significant decrease in strength was observed at a higher temperature (e.g., 600 °C). For thermal load at 250 °C, the average degradation in strength of mortar mixes made with 100% NS, 50% WSS, 100% WSS, 50% WCB, and 100% WCB was 16, 18, 15, 22 and 23%, respectively, for tensile strength, and 20, 18, 21, 20 and 28%, accordingly, for flexural strength, compared to the strength of unheated mortar specimens. The degradation of strength at this temperature could be attributed to the increase in porosity [30] and permeability [33] in the mortar matrix by the opening of the pore systems induced by the evaporation of free water and part of the physically bound water expulsion from hardened cement paste and aggregates. This behavior is in agreement with the literature [33,34,35]. The authors found a significant increase in permeability at temperatures from 80 to 120 °C.

Moreover, the vapor pressure in the pores would be significantly higher at this temperature, leading to higher tensile stress (i.e., higher permeability in the mortar matrix due to higher cracks), resulting in lower strength. The literature showed that the vapor pressure in the pores could reach up to 4.5 MPa at 250 °C [31]. It was also reported that the fire spalling of concrete due to higher vapor pore pressure and stresses occurred at around 130–170 °C [35]. However, at the same time, thermal stresses would be significantly higher in the mortar matrix. As a result, severe cracking (i.e., higher permeability) would occur inside the mortar specimens due to the combined effect of pore pressure and thermal stresses, which should be the main reason behind the lower residual strength of mortar mixes at this temperature.

**Figure 10:**
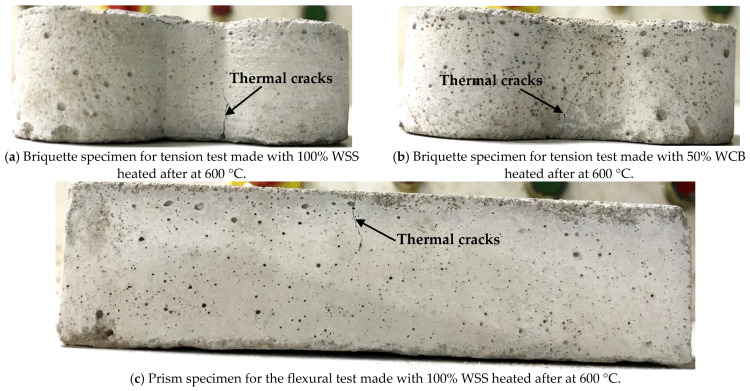

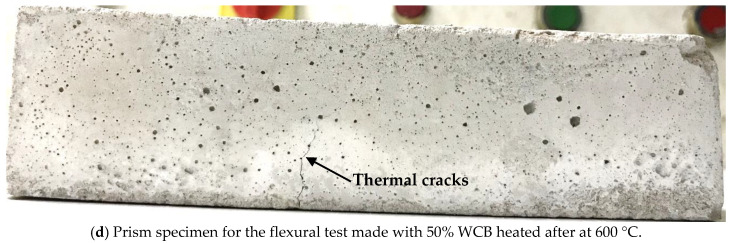
Images of mortar specimens after exposure to 600 °C: tensile test specimens (**a**,**b**) and flexural test specimens (**c**,**d**).

At higher temperatures (e.g., 400 and 600 °C), all the mortar specimens suffer more, and a faster reduction in tensile and flexural strength occurred than in the unheated specimens. For example, at 600 °C, the average degradation in strength of the mortar mixes made with 100% NS, 50% WSS, 100% WSS, 50% WCB and 100% WCB was 52, 56, 60, 66 and 73%, respectively, for the tensile strength, and 60, 66, 70, 70 and 75%, accordingly, for the flexural strength, compared to the strength of unheated mortar specimens. This significantly higher reduction in the strength of mortar mixes revealed that the materials were no longer homogeneous in their matrix for these high temperatures. This higher reduction in strength could be linked to the remarkable loss of water from the gel pores, increasing the dehydration rate, the decomposition of cement hydrated minerals such as calcium-silicate-hydrate (CSH) and calcium-aluminium-silicate-hydrate (CASH) and the coefficient of the thermal expansion of cement paste and aggregate [33,34], which leads to severe cracks (Figure 10) in the mortar matrix, resulting in lower strength of the mortar. Furthermore, the reduction in the strength could also be due to moisture absorption from the atmosphere and the rehydration of calcium oxide (CaO) during cooling, leading to an increase in the volume of the mortar specimens (i.e., higher cracking, Figure 10).

Regarding the effect of material type, it was found that the mortar made with WSS and WCB suffered more than the material made with NS at elevated temperatures. The maximum reduction was observed for the mortar made with WCB. The remaining strength of the mortar made with 100% NS, 100% WSS and 50% WCB after exposure to 600 °C was 48, 40 and 34%, respectively, for tensile strength, and 40, 30 and 30%, accordingly, for flexural strength, compared to the unheated specimens. Two main reasons could explain the higher strength reduction in WSS and WCB than NS: (i) the significantly higher initial tensile and flexural strength of mortar made with 100% WSS (76% for tensile and 68% for flexural strength higher than 100% NS) and 50% WCB (25% for tensile and 37% for flexural strength higher than 100% NS) and (ii) the higher content of coarser particles (percentage materials retained on 2.36 and 4.75 mm sieves were 0.75 and 5.78 for NS, 4.2 and 27 for WSS and 7.0 and 35.33 for WCB, Figure 2) of WSS and WCB than NS. The higher initial strength (i.e., lower porosity and denser matrix, Figure 6) of mortar made with WSS and WCB could induce a significantly steeper pressure gradient and higher vapor pore pressure followed by the higher tensile stresses in the matrix, resulting in lower tensile and flexural strength due to higher cracks in the mortar matrix. The evidence of these cracks in both tensile and flexural test specimens after exposure to 600 °C was observed for WSS and WCB (Figure 10).

As the mortar made with WSS and WCB contained a higher amount of coarser particles than NS (Figure 2), a significantly higher permeability could occur in the mortar matrix due to the thermal cracks induced by the thermal incompatibility between the aggregates and cement paste. The literature shows that the cement paste shrinks at high temperatures due to dehydration; on the other hand, the aggregates expand due to the positive thermal expansion [30,33,34]. Therefore, the tensile stresses could occur in the mortar matrix due to their different behavior, leading to higher thermal cracks at the ITZ, through the aggregates and the mortar matrix. These thermal cracks (i.e., higher permeability) would increase with the increasing amount of coarser particles in the mortar (Figure 10). These higher thermal cracks of the mortar made with WSS and WCB should be one of the main reasons for the lower strength than NS. This behavior is in agreement with the literature [22,32,36]. It was shown that a significantly higher permeability occurred when heated at a higher temperature (e.g., 300 °C) for the mix made with coarser particles (size of aggregate is around 5 mm) than the mix containing a small-sized aggregate of 0.6 mm [19]. Miah et al. [32] showed that the mortar containing NS exhibited lower strength loss at elevated temperatures (e.g., 600 °C) compared to the mortar made with recycled iron powder (RIP), which was coarser than NS (fineness modus of NS and RIP was 2.86 and 3.15, respectively). Roy et al. [22] investigated the residual mechanical properties (compressive strength, elastic modulus, flexural strength and tensile fracture energy) of concrete containing EAF slag as a partial/complete replacement for natural sand at elevated temperatures (200–1000 °C). At higher temperatures (> 500 °C), the reduction in mechanical strength was greater for concrete made with fine slag aggregate than NS due to the higher internal cracks and interconnected surface cracks in slag concrete than in NS concrete. These results are in good agreement with the present study.

## 4. Conclusions

This research investigates the tensile and flexural strength of mortar using waste steel slag (WSS) and waste clay brick (WCB) as a partial/complete replacement for natural sand (NS) at ambient temperatures and after exposure to elevated temperatures. The following conclusions are drawn based on the findings of this research below:

1. At normal temperature, both tensile and flexural strength increased with the increasing percentage of WSS (50–76% for tensile and 39–68% for flexural strength), while the strength increased for 50% WCB (16–25% for tensile and 18–37% for flexural strength) and decreased for 100% WCB compared to 100% NS.
2. The long-term strength of mortar made with WSS and WCB was better than that made with NS due to the micro-filler and possible activation of the pozzolanic reaction due to the sufficient specific surface area WSS and WCB permitting the pozzolanic reaction.
3. The dry density of the mortar mixes decreased with an increase in thermal load, and the drop was higher at the higher temperature (e.g., at 600 °C) and at 100% WCB. These results agree with the higher mass loss at the highest temperature and higher content of WCB.
4. The tensile and flexural strength of all mortar mixes decreased with increasing exposure temperature, with a significant effect at higher temperatures. The residual strength of mortar made with 100% NS, 100% WSS and 50% WCB after exposure to 600 °C was 48, 40 and 34%, respectively, for tensile strength, and 40, 30 and 30%, for flexural strength, compared to the strength of unheated specimens.
5. Natural sand can be entirely replaced by WSS and by 50% WCB.

## Figures and Tables

**Table 1:** Chemical composition of cement, NS, WSS and WCB.

Chemical Composition	Cement (%)	NS (%)	WSS (%)	WCB (%)
SiO_2_	24.90	75.45	26.18	60.43
Fe_2_O_3_	3.96	5.08	44.39	14.27
Al_2_O_3_	7.52	7.62	4.94	9.96
K_2_O	1.00	4.62	0.56	5.23
CaO	53.43	3.13	4.94	4.18
TiO_2_	1.18	0.69	1.73	1.81
MgO	2.52	1.26	0.47	1.69
Na_2_O	0.27	1.72	0.45	0.90
SO_3_	4.77	-	0.43	0.57
MnO	0.07	0.08	12.90	0.30
P_2_O_5_	0.21	0.17	0.08	0.24
ZrO_2_	0.01	-	0.11	0.05
SrO	0.08	0.04	0.09	0.05

**Table 2:** Mix design of mortar mixes (kg/m^3^).

Mix ID	Cement	Fine Aggregates			Water
		NS	WSS	WCB	
Control	544.7	1361.8	-	-	272.4
50% WSS	544.7	680.9	861.7	-	272.4
100% WSS	544.7	-	1723.5	-	272.4
50% WCB	544.7	680.9	-	452.1	272.4
100% WCB	544.7	-	-	904.3	272.4

## Data Availability

The data presented in this study are available on request from the corresponding author.
